# PAIS: paracetamol (acetaminophen) in stroke; protocol for a randomized, double blind clinical trial. [ISCRTN 74418480]

**DOI:** 10.1186/1471-2261-5-24

**Published:** 2005-08-19

**Authors:** Eric J van Breda, H Bart van der Worp, H Maarten A van Gemert, Ale Algra, L Jaap Kappelle, Jan van Gijn, Peter J Koudstaal, Diederik WJ Dippel, the PAIS investigators

**Affiliations:** 1 Erasmus Medical Center Rotterdam, the Netherlands; 2 University Medical Center Utrecht, the Netherlands; 3 Meander Medical Center, the Netherlands

## Abstract

**Background:**

In patients with acute stroke, increased body temperature is associated with large lesion volumes, high case fatality, and poor functional outcome. A 1°C increase in body temperature may double the odds of poor outcome. Two randomized double-blind clinical trials in patients with acute ischemic stroke have shown that treatment with a daily dose of 6 g acetaminophen (paracetamol) results in a small but rapid and potentially worthwhile reduction of 0.3°C (95% CI: 0.1–0.5) in body temperature. We set out to test the hypothesis that early antipyretic therapy reduces the risk of death or dependency in patients with acute stroke, even if they are normothermic.

**Methods/design:**

Paracetamol (Acetaminophen) In Stroke (PAIS) is a randomized, double-blind clinical trial, comparing high-dose acetaminophen with placebo in 2500 patients. Inclusion criteria are a clinical diagnosis of hemorrhagic or ischemic stroke and the possibility to start treatment within 12 hours from onset of symptoms. The study will have a power of 86% to detect an absolute difference of 6% in the risk of death or dependency at three months, and a power of 72% to detect an absolute difference of 5%, at a 5% significance level.

**Discussion:**

This is a simple trial, with a drug that only has a small effect on body temperature in normothermic patients. However, when lowering body temperature with acetaminophen does have the expected effectiveness, 20 patients will have to be treated to prevent dependency or death in one.

## Background

In patients with acute stroke, increased body temperature is associated with high case fatality and poor functional outcome [1]. In the observational Copenhagen Stroke study, a 1°C increase in body temperature measured within 12 hours after stroke onset doubled the odds of poor outcome. The relation between body temperature and outcome was not affected by severity of symptoms at admission, lesion volume, or stroke type [1-6]. This suggests that even a small reduction in body temperature in acute stroke could improve outcome.

### Phase II studies

We conducted two randomized, placebo-controlled clinical trials to study whether early treatment with acetaminophen reduces body temperature in patients with acute ischemic stroke confined to the carotid territory. In the first trial, seventy-six patients were randomized to daily treatment with either 3000 or 6000 mg acetaminophen, or placebo. In the high-dose group this resulted in a 0.4°C (95% CI: 0.1 to 0.7°C) lower body temperature than placebo treatment at 24 hours after onset [7]. The second trial was conducted to study the effect of ibuprofen and to confirm the effect of high-dose acetaminophen on body temperature. Seventy-five patients with acute ischemic stroke of the anterior circulation were randomized to daily treatment with either 2400 mg ibuprofen, 6000 mg acetaminophen, or placebo during 5 days [8]. Treatment with high-dose acetaminophen resulted in a 0.3°C reduction (95% CI: 0.0 to 0.6°C) in body temperature at 24 hours compared to treatment with placebo [9]. A pooled analysis of the data from both studies showed that a significant decrease of body temperature occurred within 4 hours after start of treatment with high-dose acetaminophen [10]. These studies suggest that high-dose acetaminophen can induce a small, but early reduction in body temperature in patients with acute stroke.

### Pharmacological properties and toxicity of acetaminophen

Acetaminophen is a potent inhibitor of prostaglandin production within the central nervous system. This presumably accounts for its analgesic and antipyretic properties. It is rapidly absorbed through the gastrointestinal tract and uniformly distributed through most body fluids. After oral administration peak plasma levels are reached after 30 minutes to 1 hour [11]. Rectal administration may lead to a slightly slower absorption, but effective plasma levels are reached after approximately one hour [12]. Acetaminophen is mainly conjugated in the liver, and then excreted in the urine. Plasma half-life is 1.5 to 3 hours. Following a dose of 140 mg/kg bodyweight or more, the glucuronide pathway may become saturated, and (hepato-) toxicity may result. In the above-mentioned pilot trials, acetaminophen in a dose of 6 g/day was not associated with hepatotoxicity or other side effects [7,9].

### Aim and purpose of the study

In guidelines for the treatment of acute ischemic or hemorrhagic stroke, antipyretic therapy is recommended in patients who develop fever [13,14]. This recommendation is based on expert opinion and observational studies but not on randomized clinical trials. Because observational studies suggest that every decrease in body temperature, regardless of the initial body temperature, is potentially benificial, antipyretic therapy may even improve outcome in patients without fever. The aim of PAIS is to test whether early antipyretic treatment with acetaminophen in a daily dose of 6000 mg for a period of 3 days will improve functional outcome in patients with acute stroke, even if they are normothermic.

## Methods

### Study design

PAIS is a multicenter, double-blind, placebo-controlled clinical trial that aims to include 2500 patients with acute stroke. The statistical analysis will be performed on an intention-to-treat basis [15]. Publication of the study results will be on behalf of the PAIS investigators.

### Study medication

Patients will be treated for 3 days with acetaminophen in a daily dose of 6 times 1000 mg, or matching placebo. The study medication will be administered through identical tablets. The study medication will be provided in white paper boxes, numbered consecutively with a medication number. Each box contains 40 identical tablets of acetaminophen 500 mg, or placebo, and one suppository of acetaminophen 1000 mg, or placebo. Every gift of medication consists of 2 tablets except the first gift, which can be given as a suppository. The suppository can be administered at the discretion of the treating physician, for example when a patient has swallowing difficulties but has not yet been given a nasogastric tube.

### Inclusion- and exclusion criteria

Inclusion criteria are a clinical diagnosis of ischemic stroke or primary intracerebral hemorrhage, the possibility to confirm the diagnosis with CT or MRI within 24 hours after inclusion in the study, the possibility to start treatment within 12 hours from onset of symptoms (for patients who noticed symptoms after waking from sleep, the time last seen well is taken as the time of onset of symptoms), age over 18 years, and signed informed consent.

Exclusion criteria are body temperature of less than 36.0°C or more than 39.0°C, a history of liver disease or elevated liver enzymes (ASAT, ALAT, AP, or gamma-GT) to more than twice the upper limit of normal, a history of alcohol abuse, hypersensitivity to acetaminophen, death appearing imminent at the time of inclusion, and any pre-stroke impairment that has led to dependency (modified Rankin scale (mRS) > 2) and therefore interferes with the assessment of functional outcome.

### Randomization and treatment schedule

Patients will be assigned to treatment with acetaminophen 1000 mg, 6 times daily, or to matching placebo, for three days. Treatment assignment will be random. The study medication will be packed in separate boxes with a unique number. The study number is printed on an adhesive label, to be put on the inclusion form.

The study numbers correspond to those on a computer-generated list with the assigned treatment. An independent statistician, who is not involved in the study, will provide the list. The pharmacist of each center will receive a list that indicates the treatment allocation for each randomized patient.

### Safety concerns and adverse events

Local investigators are advised to be careful not to overlook infections in patients who are treated according to the study protocol and might be treated with acetaminophen. Therefore, treating physicians are advised to lower their threshold for clinical suspicion of infection, and to start diagnostic studies and antibiotic treatment earlier than usual.

Serious adverse events will be reported by the local investigator to the PAIS trial office and include any infection that leads to prolonged hospital stay or is life threatening, death from any cause, liver failure, and gastro-intestinal hemorrhage that occurs during hospitalization and within the first 14 days after randomization.

### Outcomes

The primary outcome measure will be functional outcome, as determined by the score on the modified Rankin scale (mRS) at 3 months [16]. Outcome will be dichotomized and defined as good (mRS 0 to 2) or poor (mRS 3 to 6). Other outcome measures will include an alternative dichotomization of the mRS (0 to 3 versus 4 to 6), the score on the Barthel index (BI) at 3 months [17], and body temperature at 24 hours from start of treatment. In addition, quality of life will be measured at three months with the EuroQol-5D [18,19].

## Design

### Time path

The study will run for four years. Patient inclusion will continue during the first 3.5 years. Three months will be needed to conclude the follow-up, and three months to run the final analysis and prepare the final report. In order to include 2500 patients in the study within the given timeframe, an annual recruitment rate of more than 700 patients will have to be realized.

### Study activities

Day 0 is defined as the time period between onset of stroke and inclusion in the study. All baseline investigations are therefore carried out on day 0, except for the CT or MRI scans, which may be done within 24 hours after inclusion into the study. Day 1 commences directly after inclusion into the study. All time periods in the study are measured relative to the time of the start of treatment.

### Baseline data

At baseline the medical history, including previous strokes or TIAs, will be assessed and a general and neurological examination will be carried out. The National Institutes of Health stroke scale (NIHSS) is used to assess stroke severity at inclusion in the study [20]. Laboratory investigations will include a full blood count, glucose, electrolytes, creatinine, and liver enzymes: ALAT, ASAT, AP, and gamma-GT. A brain CT or MRI will be done within 24 hours after inclusion in the study. Body temperature (tympanic or rectal) will be measured at inclusion, and 24 hours later. In each individual patient, the mode of thermometry at 24 hours will be similar to that at baseline.

After completing the one-page inclusion form, the local investigator will send it by fax to the trial office. The data will be automatically added to the study database by means of optical character recognition soft- and hardware (Teleform, Verity Inc. Sunnyvale, U.S.A.). All data entries will be verified by a study assistant. The trial coordinator will compare the data provided by the local investigators with those in the source documents in a random sample of at least 10% of the patients.

### Day 14 or discharge

After 14 days or at discharge, if earlier, the discharge destination, the number of remaining tablets and suppositories, and functional status (BI) will be assessed. Based on the neurological examination and the results of CT, ECG, duplex, and other studies, etiological stroke type will be assessed according to the TOAST classification [21]. Serious adverse events that occurred during 14 days after start of treatment will be recorded.

### Three month follow-up

Functional outcome at three months (mRS, BI) and quality of life (EuroQol-5D) will be assessed by telephone interview with the patients themselves or their caregivers. The telephone interview will be conducted by the central trial office.

### Data analysis

The data will be analyzed on an intention-to-treat basis. In the analysis the occurrence of the primary outcome (mRS>2) will be compared between the two treatment arms by computing the relative risk, expressed as a risk ratio with a 95% confidence interval. In order to increase the power of the study, adjustments will be made with multiple logistic regression for any imbalance in the following prognostic variables: time since onset, baseline temperature, stroke severity, stroke type (hemorrhagic versus ischemic), ischemic stroke subtype (lacunar versus non-lacunar), and thrombolytic therapy, as suggested by Hernandez [22].

The treatment effect will be evaluated in specific subgroups, i.e. in patients treated early (i.e. within 9 hours from onset), in patients with ischemic stroke (as opposed to hemorrhagic stroke), and in patients with non-lacunar ischemic stroke.

### Interim analysis of safety and effectiveness

During the period of recruitment into the study, every year interim analyses of in-hospital mortality and of any other information that is available on major outcome events including serious adverse events believed to be due to treatment will be supplied, in strict confidence, to the chairman of the data monitoring committee, along with any other analyses that the committee may request. In the light of these analyses, the data monitoring committee will advise the chairman of the steering committee if, in their view, the randomized comparisons in PAIS have provided both: 1) proof beyond reasonable doubt that for all, or for some, specific types of patients, one particular treatment is clearly indicated or clearly contra-indicated in terms of a net difference in mortality, and 2) evidence that might reasonably be expected to influence materially the patient management of the many clinicians who are already aware of the results of other main trials. The steering committee can then decide whether to modify intake to the study (or to seek extra data). Unless this happens, however, the steering committee, the collaborators, and the central administrative staff (except those who produce the confidential analyses) will remain ignorant of the interim analyses. Appropriate criteria of "proof beyond reasonable doubt" cannot be specified precisely, but some members of the committee have expressed sympathy with the view that a difference of at least 3 standard deviations in an interim analysis of a major outcome event may be needed to justify halting, or modifying such a study prematurely. If this criterion were to be adopted, it would have the practical advantage that the exact number of interim analyses would be of little importance, and so no fixed schedule is proposed.

Collaborators, and all others associated with the study, may write through the PAIS trial-office in Rotterdam to the chairman of the data monitoring committee, drawing attention to any worries that they may have about the possibility of particular side-effects, or about particular categories of patients requiring special consideration, or about any other matters that may be relevant [23].

### Sample size

In the Copenhagen Stroke Study, the odds ratio of poor outcome increased by a factor of 2.2 (95% CI: 1.4 to 3.5), with each degree Celsius increase in body temperature [5,24]. We expect that 50% of the patients assigned to placebo will have a poor outcome at three months [7,9,10]. A relative odds reduction with a factor of 2.2 per degree Celsius of body temperature would imply an absolute reduction in poor outcome of 18.75%. A 0.3°C reduction in body temperature would then theoretically lead to a 6% absolute or 12% relative reduction of poor outcome. Two thousand five hundred patients (1250 in each arm) will provide a power of 86% to detect at least such an effect, and a power of 72% to detect an absolute reduction of 5%.

## Discussion

Several studies have demonstrated a strong relationship between an increased body temperature in the first hours after stroke and poor functional outcome [3,5,24-26]. To date, it is still unclear whether this relationship is causal. Some authors suggest that increased body temperatures after stroke are just an epiphenomenon of extensive cerebral damage and, thereby, of poor outcome. In a recent study of 725 consecutive patients admitted within 6 hours from the onset of acute ischemic stroke, no relation was found between initial body temperature and outcome [27]. The authors used a rather insensitive method of statistical analysis (Spearman correlation and comparison of median modified Rankin scale scores with a nonparametric test). All patients with a body temperature of 37.0°C were treated with acetaminophen. Consequently, the results of this study should be interpreted with care.

In the Copenhagen Stroke study, the relationship between body temperature and outcome remained present after adjustment for initial stroke severity [5]. Furthermore, early body temperature measurements (within the first 6 hours) seemed to be more strongly related to outcome than later measurements [24]. This suggests that the relationship with poor outcome is not confounded by the occurrence of secondary infections, such as pneumonia or urinary tract infection, because these usually appear later in the course of the disease. Arguments for a causal relationship stem from the observation that the effect of body temperature on outcome is independent of the size of the brain lesion and from the beneficial effect of temperature-lowering treatment on infarct volume in animal models. Animal studies have demonstrated that higher body temperatures may worsen ischemic damage through an increase of blood-brain-barrier permeability and increased metabolic demands, resulting in acidosis and higher levels of deleterious excitatory amino-acids [28]. In a recent meta-analysis of controlled animal studies on the effect of hypo- and hyperthermia in focal cerebral ischemia, Miyazawa showed that hyperthermia increases infarct volume whereas hypothermia reduces infarct volume [29]. These reproducible observations from observational clinical studies and animal experiments strongly suggest that hypothermia may be a potent neuroprotective intervention, but this has never been studied in adequately powered clinical trials.

Randomized clinical trials of hypothermia in brain injury and hypoxic brain damage have provided conflicting results. In patients with coma after closed head injury, treatment with hypothermia, with body temperature reaching 33°C within eight hours after injury, was not effective in improving outcome [30]. In patients who had been successfully resuscitated after cardiac arrest, hypothermia with body temperatures between 32°C and 34°C increased the chances of favorable outcome and reduced mortality [31,32]. Perhaps the lack of effect in traumatic brain injury can be explained by the abundant presence of direct, i.e. non-ischemic damage.

### Other methods of body temperature reduction

The feasibility of different methods of reducing body temperature in patients with acute stroke has been studied in several pilot studies.

A case-control study was conducted in 74 patients (17 cases, 56 controls) to assess the feasibility and safety of reducing body temperature to approximately 35.5°C with cooling blankets in combination with pethidine to prevent shivering [33].

Another study on the feasibility of several methods of lowering body temperature was conducted in eight patients. Two patients were treated with 1 gram acetaminophen at 4-hour intervals. Two patients were cooled with cooling blankets, in two patients sponging with 70% alcohol was applied, and 2 patients served as a control group and were only monitored. Target temperature reductions of 1°C were reached within 6 hours [34].

A study in 50 patients with a severe middle cerebral artery infarct demonstrated that body temperature can be reduced to 32 to 33°C, with the use of cooling blankets, alcohol and ice bags, under complete anesthesia [35]. The procedure had many side effects; the most frequent complications were thrombocytopenia (70%), bradycardia (62%), and pneumonia (48%).

The many complications that occurred in the study of Schwab can be explained by the fact that these patients all had severe middle cerebral artery infarcts. In the remaining studies safety concerns were less prominent.

The COOL-AID study was a randomized controlled study of endovascular cooling to 33°C compared to standard medical treatment in 40 patients with acute ischemic stroke. Shivering was suppressed by warming blankets and sedatives [36].

Most studies showed that cooling using the different methods was feasible, although it was more labor intense in the more invasive methods that induced larger body temperature decreases. However, in the COOL-AID study the feasibility was poor: 5 of the 18 treated patients did not reach the target temperature.

Mild reductions in body temperature – around 1°C – can be reached with external cooling blankets and general measures, within six hours, or more rapidly with an endovascular temperature management system. This approach requires mild sedation or morfine to reduce shivering, may be uncomfortable to patients, and is labor intensive. More aggressive approaches require anesthesia, and may induce increased risk of pulmonary- and other complications. This suggests that there is a need for a simple, medical intervention that may reduce body temperature to a lesser extent, but is cheap and safe.

### Ethical considerations

Observational studies have shown an association between an increased body temperature and a poor outcome. Several national and international guidelines suggest that raised body temperature in stroke patients should be treated [13]. However, the efficacy of temperature-lowering treatment to improve functional outcome has not yet been demonstrated in randomized trials [37]. This dilemma is the rationale for PAIS.

### PAIS: a simple trial

PAIS is a large, randomized, multi-center clinical trial. To keep the threshold for including patients low, the study design has been kept simple. The amount of data to be gathered is therefore limited; it consists of two one-page forms, one to be filled out at inclusion and one to be filled out at discharge. The local investigator will keep a log of randomized patients. The 3-month follow-up will be conducted by telephone from the central trial office. New centers are welcome to join the trial.

## Conclusion

The PAIS trial will test the efficacy of a modest temperature reduction by high-dose acetaminophen to improve functional outcome after stroke. The treatment strategy tested is cheap and safe. When lowering body temperature with acetaminophen will have the expected effectiveness, 20 patients will have to be treated to prevent functional dependence or death in one

## Abbreviations

ASAT – aspartate aminotransferase

ALAT – alanine aminotransferase

AP – alkaline phosphatase

BI – Barthel Index

GT – glutamyl transpeptidase

CRP – C-reactive protein

CT – computed tomography

mRS – modified Rankin Scale

MRI – magnetic resonance imaging

NIHSS – National Institutes of Health Stroke Scale

PAIS – Paracetamol (Acetaminophen) In Stroke

TOAST – Trial of ORG 10172 in Acute Stroke Treatment

## Competing interests

The author(s) declare that they have no competing interests.

## Financial support

This study is financially supported by the Netherlands Heart Foundation; Grant no 2002 B148.

## Authors' contributions

All authors contributed equally to this article.

## Pre-publication history

The pre-publication history for this paper can be accessed here:


