# Correction: PAIS: paracetamol (acetaminophen) in stroke; protocol for a randomized, double blind clinical trial. [ISCRTN74418480]

**DOI:** 10.1186/1471-2261-8-29

**Published:** 2008-11-04

**Authors:** Heleen M den Hertog, H Bart van der Worp, H Maarten A van Gemert, Ale Algra, L Jaap Kappelle, Jan van Gijn, Peter J Koudstaal, Diederik WJ Dippel

**Affiliations:** 1 Department of Neurology, Erasmus MC University Medical Center, Rotterdam, the Netherlands; 2 Department of Neurology, Rudolf Magnus Institute of Neuroscience, University Medical Center Utrecht. Utrecht, the Netherlands; 3 Department of Neurology, Meander Medical Center Amersfoort, the Netherlands; 4 Department of Neurology and Julius Center, University Medical Center Utrecht, and department of Clin Epidemiology, Leiden University Medical Center, the Netherlands

## Abstract

**Background:**

The Paracetamol (Acetaminophen) In Stroke (PAIS) study is a phase III multicenter, double blind, randomized, placebo-controlled clinical trial of high-dose acetaminophen in patients with acute stroke. The trial compares treatment with a daily dose of 6 g acetaminophen, started within 12 hours after the onset of symptoms, with matched placebo. The purpose of this study is to assess whether treatment with acetaminophen for 3 days will result in improved functional outcome through a modest reduction in body temperature and prevention of fever.

The previously planned statistical analysis based on a dichotomization of the scores on the modified Rankin Scale (mRS) may not make the most efficient use of the available baseline information. Therefore, the planned primary analysis of the PAIS study has been changed from fixed dichotomization of the mRS to a sliding dichotomy analysis.

**Methods:**

Instead of taking a single definition of good outcome for all patients, the definition is tailored to each individual patient's baseline prognosis on entry into the trial.

**Conclusion:**

The protocol change was initiated because of both advances in statistical approaches and to increase the efficiency of the trial by improving statistical power.

**Trial Registration:**

Current Controlled Trials [ISCRTN74418480]

## Background

The Paracetamol (Acetaminophen) In Stroke (PAIS) Study is a phase III multicenter, double blind, randomized, placebo-controlled clinical trial of high-dose acetaminophen in patients with acute stroke. The trial compares treatment with a daily dose of 6 g acetaminophen, started within 12 hours after the onset of symptoms, with matched placebo. The purpose of this study is to assess whether treatment with acetaminophen for 3 days will result in improved long-term functional outcome through a modest reduction in body temperature and prevention of fever [1].

In the original protocol, the primary outcome measure is a dichotomized score on the modified Rankin Scale (mRS) [2] assessed at 3 months from onset of symptoms, with good functional outcome defined as a score of 0–2 and poor functional outcome as a score of 3-death.

The common approach of dichotomizing the mRS, an ordinal outcome scale, has several disadvantages. First, it may not correspond with everyday clinical practice. Dichotomized outcome analyses convert ordinal scales into binary outcome measures. Most treatment strategies tested in acute stroke trials are not expected to be completely curative, but to lead to improvement. Therefore, it is also informative to show that treatment moves patients from severe to moderate disability or from moderate disability to recovery, and not only to demonstrate differences in the numbers of patients with a good or poor functional outcome. Secondly, dichotomization may limit statistical power. The sample size calculation used for the PAIS study assumes that each patient in the placebo group has a 50% probability of poor outcome at 3 months [1]. In practice, there will be substantial prognostic heterogeneity because of differences in baseline variables between patients. If this is not taken into account, the chance of finding a true treatment effect may be reduced. Thirdly, by assessing only a single health state transition, investigators may be forced to discard a substantial amount of outcome information. This may lead to underestimation of treatment benefit or harm.

Because of these disadvantages of dichotomization, new approaches to outcome analysis have been proposed and tested in acute stroke trials [3-7]. These novel outcome analyses consider the full range of the ordinal outcome scale and lead to a single and meaningful estimate of the treatment effect. One of these new approaches is the so-called "sliding dichotomy" [5]. Other approaches are shift analysis and analyses that make use of ordinal logistic regression [3,4,7].

For the PAIS study, we decided to change the planned analysis for the primary outcome measure from a fixed dichotomy to a sliding dichotomy analysis. This protocol change was initiated because of both advances in statistical approaches and to increase the efficiency of the trial by improving statistical power.

## Methods

Instead of taking a single definition of good outcome for all patients, the definition is tailored to each individual patient's baseline prognosis on entry into the trial. The outcome of an individual patient is thus regarded as good or poor depending on what would have been expected based on the severity of stroke and other prognostic factors.

Blinded data from the PAIS study [1] were used to illustrate this procedure of the sliding dichotomy analysis (Figure 1). With logistic regression, a prognostic index to estimate the probability of good outcome (defined as mRS < 3) was generated on basis of blinded observations in the PAIS study. The prognostic index included age, sex, stroke severity (according to the NIHSS), previous stroke, stroke type (hemorrhagic or ischemic), and diabetes mellitus.

**Figure 1:**
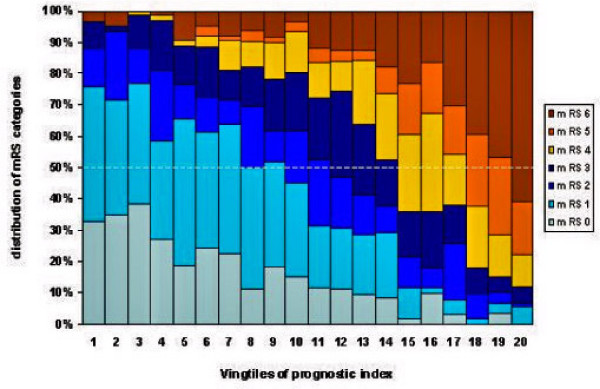
**Distribution of predicted outcome scores on the modified Rankin Scale**[2]**at three months over ordered vingtiles of a prognostic index for good outcome in the PAIS trial**[1]. Six bands were defined in such a way that the median of the mRS in that band was equal to the next score on the mRS (1–6). Cut points were taken at quantile 7/8, 11/12, 14/15, 17/18, and 19/20.

The study population was then divided into 20 quantiles (vingtiles) of the baseline prognostic index. The distribution of the mRS scores at three months was estimated for each vingtile of the prognostic index. For each vingtile the distribution of outcome mRS is given in Figure 1. Thereafter, 6 bands (clusters of vingtiles) were defined in such a way that the median of the mRS in that band would be equal to the next score on the mRS (1–6). Cut points would thus be taken at vingtiles 7/8, 11/12, 14/15, 17/18, and 19/20. The primary effect estimate will be the odds ratio of improvement, i.e. a lower score than the median, for patients in a particular band. For example, for a patient of whom the prognostic risk is in the 12^th ^vingtile, the median predicted outcome mRS is 3 and an outcome mRS < 3 would be taken as good outcome (Figure 1). This varying bandwidth makes more efficient use of the data than an approach based on tertiles or quartiles, as originally proposed [5]. Note that the choice of cut point is completely independent of the treatment effect, as the data are blinded.

Although the definition of the new primary outcome is based on prognostic indicators at baseline, imbalances in these and other prognostic factors between the treatment groups may still confound the assessment of treatment effect. Adjustments will therefore be made with a multiple logistic regression model that includes time since stroke onset, baseline temperature, stroke severity (as assessed with the NIHSS), stroke type (hemorrhagic versus ischemic), ischemic stroke subtype (lacunar versus non-lacunar) and thrombolytic therapy, as proposed previously, [1] as well as for age, sex, previous stroke, atrial fibrillation and diabetes mellitus.

Secondary effect measures will include an estimate of the odds ratio for improvement, estimated by means of ordinal logistic regression analysis.

We will also estimate the adjusted odds ratio for the former primary outcome, i.e. good functional outcome, as defined as a score ≤ 2, and alternative dichotomizations of the mRS (0 to 3 versus 4 to 6). As reported before [1], the score on the Barthel index at 3 months, body temperature at 24 hours from start of treatment, and quality of life at three months as assessed with the EuroQol-5D will also be assessed.

## Discussion

Several approaches to outcome analysis have recently been applied in acute stroke trials, including shift analysis [6], a rank test of original ordinal data, ordinal logistic regression [3], and the concept of a sliding dichotomy [5].

Analyzing the full range of ordinal data for functional outcome has been shown to be more statistically efficient than collapsing data [3]. A recent study compared statistical approaches to the analysis of differences in functional outcome as measured on ordinal scales. The authors concluded that methods that made use of the full range of ordinal outcomes were more sensitive to treatment effects. However, in this study methods that incorporate baseline prognostic information, such as the sliding dichotomy, could not be evaluated [3].

Shift analysis [6] is not a formal test, but a calculus to estimate the proportion of patients moving from one category on the ordinal scale to the next. This can be a useful measure to gain insight in the size of a treatment effect. A disadvantage of shift analysis is that the uncertainty concerning the effect estimate cannot be quantified, as in statistical effect estimation.

The term 'shift test' is used to designate the Mantel-Haenszel (CMH) test that compares two ordered outcome distributions after adjusting for one or more baseline variables. The test provides a p-value, but not an effect estimate. The Van Elteren variant of the test was employed in the analysis of the results of the GAIN trial [8] and in a post-hoc analysis of NINDS and ECASS-2 trials [9] Ordinal logistic regression was used to estimate an effect size with its corresponding 95% confidence interval.

Classic statistical methods to compare distributions on an ordinal scale require rank tests. Translation of the results of a rank test (a p-value) to an estimate of the treatment effect is not straightforward; this may be done either by shift analysis (with the aforementioned drawbacks) or by ordinal logistic regression. This analysis does not make use of the available baseline information. Ordinal logistic regression may be used to estimate treatment effects in studies with ordered outcomes. The relative risk of a transition is estimated as an odds ratio. An assumption is that any treatment effect is similar across outcome levels, i.e. the odds of moving from mRS level 3 to 2 are similar to the odds of moving from level 5 to 4. In the Optimizing Analysis of Stroke Trials (OAST) study, a study to assess which statistical approaches are most efficient in analyzing outcomes from stroke trials, the assumption of proportional odds was not met in 8 of the 55 datasets according to the authors, but they did not specify how they tested for this assumption [3]. Furthermore, this method might be inefficient when treatment effects cluster at one transition. When this approach was tested in a large dataset of patients with severe head injury, it did not perform better than the sliding dichotomy [5]. Effect estimates should be meaningful from a clinical point of view. In ordinal logistic regression, meaningful interpretation is hampered by the point that moving 1 category up on the mRS may have a different clinical interpretation when it concerns low mRS scores, compared to high mRS scores.

In our view, these are important arguments for not using ordinal regression in the *primary *outcome analysis.

An advantage of the sliding dichotomy approach is that it makes the least assumptions about the type of patients who will be included in the study, the type of outcome they will experience, and the treatment effect pattern the treatment strategy under study will exert. It provides a simple outcome measure that is relatively easy to interpret, i.e. the relative risk of improvement beyond the level that could be expected from baseline prognostic information [5]. To our opinion, this approach is to be preferred in the analysis of treatment effects employing the full range of outcomes on the mRS. With the concept of sliding dichotomy, each individual patient's baseline prognosis is taken into account. This approach may be more relevant for clinical practice and may improve statistical power, as patients at the prognostic extremes have the potential to contribute to the estimation of the treatment effect.

An exact, parametric approach to sample size estimation for studies that make use of the sliding dichotomy approach for ordered categorical outcome variables is not available. Simulation studies of randomized clinical trials in traumatic brain injury that made use of the Glasgow Outcome Scale, [10] suggest that " substantial gains in statistical efficiency can be made". Either of these approaches along with adjustment for baseline covariates gave efficiency gains equivalent to reducing the required sample size by up to 50% [5,11].

We realize that this approach may also have disadvantages. Patients, care givers, and clinicians may consider the results of this way of analysis more difficult to interpret than collapsing data into a binary outcome. We wonder whether this is really the case. Taken at face value, a transition across the boundary between mRS 2 and mRS 3 does not tell us much at all about the real health benefit of a treatment, and a common odds ratio based on sliding dichotomy, may be in fact more informative for those who have grown accustomed to it.

## Conclusion

In summary, because of the drawbacks of dichotomization, the primary outcome analysis of the multicenter acute stroke trial PAIS has been changed from fixed dichotomy of the mRS to the sliding dichotomy analysis. It has been approved by the PAIS steering committee on April 1, 2008, before inclusion into the trial was completed. This approach may be more relevant for clinical practice and is expected to increase the efficiency of the trial by improving statistical power

## Abbreviations

NIHSS: National Institutes of Health Stroke Scale; mRS: modified Rankin Scale.

## Competing interests

The authors declare that they have no competing interests.

## Authors' contributions

All authors contributed equally to this article.

## Pre-publication history

The pre-publication history for this paper can be accessed here:

http://www.biomedcentral.com/1471-2261/8/29/prepub
